# Usenamine A: a potential therapeutic agent for rheumatoid arthritis and ankylosing spondylitis through its anti-inflammatory activity

**DOI:** 10.3389/fphar.2024.1456216

**Published:** 2024-12-03

**Authors:** Yu Jeong Lee, Zijun Li, Hyun Hee Jang, Moon-Ju Kim, Kandasamy Saravanakumar, Seung Cheol Shim, Namki Cho, Eun Jeong Won, Tae-Jong Kim

**Affiliations:** ^1^ Department of Biomedical Sciences, Graduate School of Chonnam National University, Jeollanam-do, Republic of Korea; ^2^ Department of Rheumatology, Chonnam National University Medical School and Hospital, Gwangju, Republic of Korea; ^3^ Research Institute of Pharmaceutical Sciences, College of Pharmacy, Chonnam National University, Gwangju, Republic of Korea; ^4^ Division of Rheumatology, Daejeon Rheumatoid and Degenerative Arthritis Center, Chungnam National University Hospital, Daejeon, Republic of Korea; ^5^ Department of Laboratory Medicine, Asan Medical Center, University of Ulsan College of Medicine, Seoul, Republic of Korea

**Keywords:** Usenamine A, usnea diffracta, rheumatoid arthritis, ankylosing spondylitis, anti-inflammatory effects

## Abstract

**Background:**

Usenamine A (UA) is a natural compound isolated from the lichen *Usnea diffracta*, and its therapeutic effects on rheumatic diseases are not well understood. This study aimed to evaluate the potential anti-inflammatory effects of UA and its therapeutic effects on rheumatoid arthritis (RA) and ankylosing spondylitis (AS).

**Materials and methods:**

Molecular docking was performed between the 3D structure of UA and the TNF-TNFR2 complex. Peripheral blood mononuclear cells (PBMCs) from RA and AS patients were treated with UA, and cell viability was measured using the MTS assay and flow cytometry. The in vitro effects of co-culture with UA were determined by measuring inflammatory cytokines, including IFN-γ, IL-17A, and GM-CSF, using flow cytometry and enzyme-linked immunosorbent assay (ELISA). The in vivo effects of UA were evaluated using an arthritis mouse model.

**Results:**

The docking complex of UA bound to the TNF-TNFR2 complex exhibited docking scores of −5.251 kcal/mol and −6.274 kcal/mol, confirming their active sites. UA did not affect cell viability and suppressed the production of inflammatory cytokines in the PBMCs of RA (IFN-γ, IL-17A, and GM-CSF) and AS (GM-CSF) patients. The ELISA also confirmed reduced cytokine levels in the co-culture of UA and PBMCs from RA or AS patients. In the arthritis mouse model, significantly reduced clinical and histological scores were observed in the UA treatment group.

**Conclusion:**

Our findings suggest that UA has potential as a binding target for TNF, suppresses inflammatory cytokines in PBMCs, and exhibits anti-inflammatory effects on arthritis in a mouse model.

## 1 Introduction

Rheumatic inflammatory diseases (RIDs) encompass a spectrum of conditions, including rheumatoid arthritis (RA), systemic lupus erythematosus (SLE), ankylosing spondylitis (AS), and Behcet’s disease (Moutsopoulos, 2021). Particularly noteworthy among these is the chronic and progressive natures of RA and AS, which contribute to inflammatory processes that result in joint damage and a decline in patients’ physical well-being (Barczynska et al., 2015). In RA, the joint microenvironment undergoes an upregulation of various cytokines and chemokines, such as Interleukin 1β (IL-1β), Interleukin-6 (IL-6), Interleukin-17 (IL-17A), Granulocyte-Macrophage Colony-Stimulating Factor (GM-CSF), and Tumor Necrosis Factor-α (TNF-α) (McInnes and Schett, 2007). The pathogenesis of AS is primarily associated with TNF-α, along with IL-17A, IFN-γ, and GM-CSF (Kim and Lee, 2023; Yi et al., 2023). In addressing this, current therapeutic strategies involve steroid therapy designed to suppress inflammatory cytokines, with the overarching goal of relieving pain and mitigating inflammation (Bullock et al., 2018). The advent of TNF-α inhibitors has ushered in a revolutionary change in the treatment landscape for RA and AS (Smolen et al., 2010; Choi et al., 2024). Numerous randomized clinical trials attest to the efficacy and safety of TNF-α inhibitors. However, despite these advancements, therapeutic responses remain suboptimal for many individuals with RIDs, and limited targeted therapies are available for various other RIDs (Mahieu et al., 2016; Vivino et al., 2016; Touma and Gladman, 2017). Consequently, novel approaches to drug development are imperative. Recent endeavors in this direction involve exploring natural products as a novel modality for treating autoimmune disorders. Lichen metabolites emerge as a particularly intriguing group of natural compounds garnering attention in this context.

*Usnea* is categorized as a lichen, and comprises species such as *Usnea diffracta* and *Usnea longissima* Ach (Grube et al., 2009). *Usnea*, with a widespread distribution across polar, temperate, and tropical regions, including Sichuan, Yunnan, and Tibet in China (Bao and Bau, 2013), has been utilized as traditional medicine. With a long history as a folk medicine, *Usnea* had shown diverse therapeutic effects, ranging from addressing conditions like diarrhea, stomachaches, ulcer, tuberculosis, pneumonia, and malaria to treating wounds, snake bites, coughs, and parasitic diseases. Previous studies using Usenamine A (UA) have confirmed that it exhibits excellent pharmacological activity in anti-cancer (Fang et al., 2021; Yang et al., 2023; Yang et al., 2024) and anti-viral (Li et al., 2023). Despite their relatively primitive evolutionary status, lichens boast a rich array of primary and secondary metabolites, many of which exhibit unique structures (Pazdziora et al., 2023). However, there is still no established treatment involving *Usnea*-like substances in rheumatic diseases, which are chronic inflammatory rheumatic diseases. Therefore, this study aimed to evaluate the potential of anti-inflammatory effect of UA and its therapeutic effects on RA and AS.

## 2 Materials and methods

### 2.1 Human samples

Patients who met the criteria established by the American College of Rheumatology/European League Against Rheumatism and its modifications in New York (NY) were selected. Blood samples were collected from nine RA patients and nine AS patients, and peripheral blood mononuclear cells (PBMCs) were isolated. The demographic characteristics of the AS and RA patients are shown in Table 1. The study was conducted in accordance with the Declaration of Helsinki and was approved by the Ethics Committee of Chonnam National University Hospital. Written informed consent was obtained from all participants (CNUBH-2023-019).

**TABLE 1:** Clinical characteristics of enrolled subjects.

	PBMC		
	Health controls	Ankylosing spondylitis	Rheumatoid arthritis
Total number	6	9	9
Age, mean ± SD (years)	23.8 ± 1.8	43.4 ± 13.4	53.8 ± 14.2
Male, n (%)	3 (50.0)	6 (66.6)	3 (33.3)
DAS28, mean ± SD	N.A	N.A	4.76 ± 4.78
AS-DAS, mean ± SD	N.A	4.11 ± 1.15	N.A
CRP (mg/dL), mean ± SD	N.A	1.7 ± 0.5	3.3 ± 4.9
Recent treatments			
Steroids use, n (%)	N.A	0	0 (0)
Methotrexate use, n (%)	N.A	0 (0)	9 (100)
Biologic use, n (%)	N.A	0 (0)	0 (0)

PBMC, peripheral blood mononuclear cell; SD, standard deviation; DAS, disease activity score; AS-DAS, ankylosing spondylitis disease activity score; CRP, C-reactive protein; N.A, not available.

### 2.2 Isolation of UA

To isolate UA, the ethyl acetate fraction from the aerial parts of *Usnea longissima* was subjected to silica gel column chromatography and eluted with a CH_2_Cl_2_–MeOH solvent mixture (ranging from 100:1 to 1:100; v/v), which yielded seven fractions (E1–E7). E3 was further resolved using RP C18-MPLC (20% methanol to 90% methanol). UA (purity >98%, HPLC analysis) was obtained after the separation of subfraction E3-7 using semi-preparative high-performance liquid chromatography with 70% CH_3_CN in H_2_O.

### 2.3 Molecular docking

The 3D structure of UA was generated using ChemOffice software (version 15.0, Cambridge, MA, United States). Crystal structures of the TNF-TNFR2 complex (PDB ID: 3ALQ) were obtained from the Protein Data Bank (PDB: https://www.rcsb.org/). The integrity of the protein structures was ensured by employing the clean protein protocol in BIOVIA Discovery Studio. Energy minimization of proteins and ligands was performed using the Smart Minimizer algorithm, which incorporates a spherical cutoff method. This process involved 1,000 steps of steepest descent followed by a maximum of 5,000 steps, utilizing a root mean square (RMS) tolerance of 0.1 for conjugate gradients. Post energy minimization, CDOCKER was utilized to elucidate the comprehensive molecular interactions and energies within the docking complex. Additionally, the grid box dimensions for identifying the potential binding pocket within 3ALQ were established at 86 Å, 84 Å, and 88 Å for the x, y, and *z*-axes, respectively, with the grid center designated at 24.488 Å, 14.346 Å, and 90.199 Å for x, y, and *z*-axes, respectively. The molecular simulation (MS) was applied for best docking complex using “System Builder” module of Molecular Dynamics System (Desmond/Schrödinger) (Gariganti et al., 2023).

### 2.4 Cell viability assay

The cells were seeded and treated with various concentrations of UA for the indicated durations, and cell viability was assessed using the Cell Titer 96 AQueous One Solution Reagent (G3580, Promega, United States). Following the manufacturer’s instructions, 20 μL of MTS solution was added to 100 μL of the cell culture medium and incubated at 37°C for 2–4 h. Absorbance was measured at a wavelength of 490 nm using a 96-well microplate reader (Molecular Devices, United States). Additionally, living cells were surface-stained with anti-Fixable Viability Dye-eFluor780 (65-0865-14, Invitrogen, United States), and cell viability was confirmed through flow cytometry.

### 2.5 Isolation of PBMCs

Peripheral venous blood samples were collected in heparin-containing tubes. PBMCs were isolated by density-gradient centrifugation using Ficoll-Paque Plus solution (Amersham Biosciences, Uppsala, Sweden). Freshly isolated PBMCs were suspended in a complete medium consisting of 10% Fetal Bovine Serum (Welgene, S001-01) and 1% penicillin-streptomycin solutions (100X, Welgene, LS202-02) in RPMI 1640 (Welgene, LM011-01) were used. The Cells were seeded in a 96-well plate at a density of 5 × 10^5^ cells/well.

### 2.6 Treatment with UA in human PBMCs

PBMCs in a 96-well culture plate were stimulated with Dynabeads Human T Activator CD3/CD28 (11131D, Gibco, United States) and treated with 20 μg/mL of UA, then cultured in a CO_2_ incubator at 37°C for 24 h. The cells were then stained with Pacific Blue-conjugated anti-CD4 (300521, BioLegend, United States) and anti-Fixable Viability Dye-eFuor780 (65-0865-14, Invitrogen, United States). Following this, the cells were washed, fixed, permeabilized with Perm/Wash buffer, FITC Mouse anti-human IFN-γ (552887, BD, United States), APC-conjugated anti-IL-17A (512334, BioLegend, United States), and PerCP-Cy5.5-conjugated anti-GM-CSF (502312, BioLegend, United States) antibody and analyzed with FlowJo Software (BD, United States). The levels of inflammatory cytokines in *ex vivo* culture supernatants from PBMCs were measured using ELISA assays such as Human GM-CSF ELISA (88-8337, Invitrogen, Austria), Human IFN-γ ELISA (88-7314, Invitrogen, Austria), Human TNF-α ELISA (88-7344, Invitrogen, Austria), Human IL-17A ELISA (88-7176, Invitrogen, Austria), and Human IL-6 ELISA (88-7066, Invitrogen, Austria). Optical density (OD) was recorded by a SpectraMax^®^ M2 (Molecular Devices Corp., United States) set at 450 nm.

### 2.7 Experimental arthritis mouse model

All experiments were performed with approval of the Institutional Animal Care and Use Committee (CNU IACUC-H-2018-35). SKG mice on a BALB/c background were purchased from CLEA Japan (Tokyo, Japan) and bred in a specific pathogen-free facility. In this study, female mice were used, and three groups were established: a negative control (n = 10 mice), a disease control group (n = 10 mice), and an UA treatment group (n = 10 mice). To induce experimental arthritis, 3 mg/kg of curdlan suspension (Wako, Osaka, Japan) was injected intraperitoneally (i.p.) to mice aged 8 weeks for both the disease control and the UA treatment group. One week after curdlan injection, the drug was diluted in water and provided to the mice. The positive control group was treated with water, and the UA treatment group was treated with 0.5 mg/kg per day. The experiment lasted a total of 7 weeks, and the mice’s clinical signs were monitored twice a week and scored by two independent observers (Figure 4A). Scores of the affected joints were summed as follows: 0 = asymptomatic, 0.1 = Swelling per toe, 0.5 = swelling of the ankle, 1 = severe swelling of the ankle. Six points were the highest possible points (Jo et al., 2021).

### 2.8 Histological analysis

After the experiment was over, the mice were sacrificed, and samples of the ankles and intestines were collected. Hematoxylin and Eosin (H&E) method was used to confirm histological differences. Specimens of the ankle and gut were obtained from mice and fixed with 10% formalin for 1 week. After fixation, specimens were decalcified in 10% formic acid with shaking at 37°C for a week and embedded in paraffin. Paraffin blocks were sectioned at a thickness of 3.5 µm and deparaffinized in neo-clear (109843, Merck, United States), hydrated with graded ethanol, and stained with Safranin O. All staining procedures followed standard protocols. The scores of the affected joints were summed according to a previous report (Lee et al., 2021). Two blinded readers performed pathological scoring.

### 2.9 Statistical analysis

Statistical analysis was performed using Prism 9.0 Software (GraphPad Software, San Diego, CA, United States). The statistical significance of differences between means was assessed using the Kruskal–Wallis test with Dunn’s multiple comparisons, Mann-Whitney test, the Wilcoxon matched-pairs signed rank test, two-way Analysis of variance (ANOVA) with Dunn’s multiple comparisons and Mann–Whitney test. For all graphs, *P*-value less than 0.05 was considered significant and marked as follows: *P < 0.05; **P < 0.01; ***P < 0.001; and ****P < 0.0001.

## 3 Results

### 3.1 Molecular docking studies of UA

**FIGURE 1:**
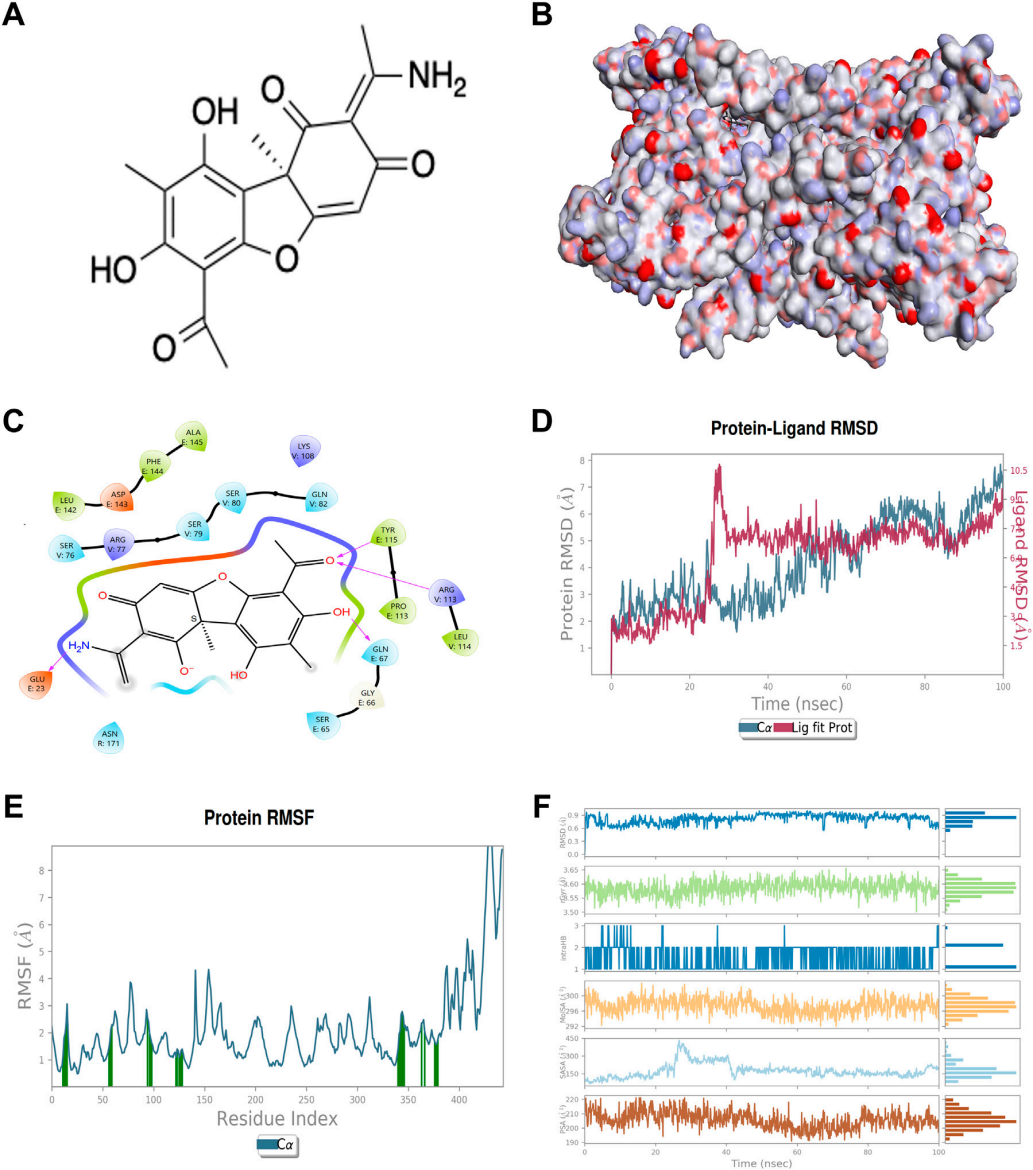
Chemical structure of Usenamine A (UA) and computational assessment of its binding interaction with the TNF-TNFR2 complex. **(A)** Chemical structure of usenamine A (UA). **(B)** Surface binding of the protein (3ALQ) **(C)** 2D view of docking complex UA-3ALQ interactions in binding pocket **(D)** RMSD trajectory of the UA-3ALQ complex during molecular simulation (100 ns). **(E)** RMSF maps of UA-3ALQ complex in molecular simulation **(F)** profile of UA molecules during the 100 ns simulation with 3ALQ protein.

The computational assessment of the binding interaction between UA and the TNF-TNFR2 complex (PDB ID: 3ALQ) was conducted. The structure of UA is shown in Figure 1A, and the surface representation of 3ALQ (receptor) highlights the complementarity of the ligand (Usenamine A) to the protein’s surface features (Figure 1B). The docking complex UA-3ALQ exhibited a docking score of −5.251 kcal/mol (Figure 1C). The molecular simulation studies were performed to demonstrate the stability of the UA-3ALQ complex through coordinates, velocities and energies were investigated through a 100 ns, with root mean square deviation (RMSD) trajectories presented in Figure 1D. After an initial equilibration phase, the RMSD values plateau, indicating a stable ligand-protein interaction. The residue flexibility, as indicated by root mean square fluctuation (RMSF) (Figure 1E), shows that certain residues exhibit greater fluctuations.

RMSD, gyration radius (rGyr), hydrogen bonding interactions (IntraHB), solvent accessible surface area (SASA), and polar surface area (PSA) elucidate different aspects of the molecular interaction of ligand. The RMSD values for UA remained under 0.6 Å throughout the simulation, indicating a stable ligand conformation within the protein binding site. An RMSD value ranging from 1 to 3 Å is acceptable for the model complex (Gariganti et al., 2023; Kim et al., 2023). This low RMSD suggests that UA maintains a consistent binding pose, aligning with findings from previous studies that stable RMSD values correlate with ligand efficacy (Fusani et al., 2020; Gariganti et al., 2023). The rGyr plot values hover around 3.55 Å, indicating a rigid conformation of UA. The molecular SASA fluctuates around 300 Å, implying that UA is conducive to maintaining structural integrity and function. PSA is closely related to the pharmacokinetic properties of the molecule, such as solubility and permeability. The PSA values are moderately fluctuating but remain within a range that suggests favorable drug-like properties for UA (Figure 1F).

Following the stability confirmation, the protein-ligand interactions of UA-3ALQ complex were analyzed (Supplementary Figure S1A, B). The interaction frequency of UA with active site amino acids in 3ALQ was scrutinized over a 100 ns molecular dynamics simulation. The intensity of interaction, as visualized through the temporal heat map, reveals the persistence and prevalence of these interactions over time, offering a deeper understanding of the binding landscape (Supplementary Figure S1A). Notably, residues such as Glu67, Ser76 and Gln82 exhibit frequent and prolonged interactions with UA, as shown by the dense clustering of marks throughout the simulation. This suggests that these residues may play a pivotal role in the binding affinity of UA to the 3ALQ protein. Such persistent interactions are indicative of strong hydrogen bonding or electrostatic interactions, which have been previously identified as critical determinants of ligand binding stability (Bitencourt-Ferreira et al., 2019). Furthermore, the interaction (ionic interactions, hydrophobic interactions, water bridges, and hydrogen bonding) were investigated for ligand and protein complex (Supplementary Figure S1B). The results revealed that the amino acid residues such as Gln21, Gly66, Gln67, Ala111, Leu142, Phe144, Ser76, Arg77, Ser79, Ser80, Gln82, and Cys115 formed the H-bonds whereas Ala111, Pro113, Tyr115, Cys78, Trp102 and Leu114 exhibited the hydrophobic interactions with UA (Supplementary Figure S1B).

### 3.2 UA has No effect on cell viability

**FIGURE 2:**
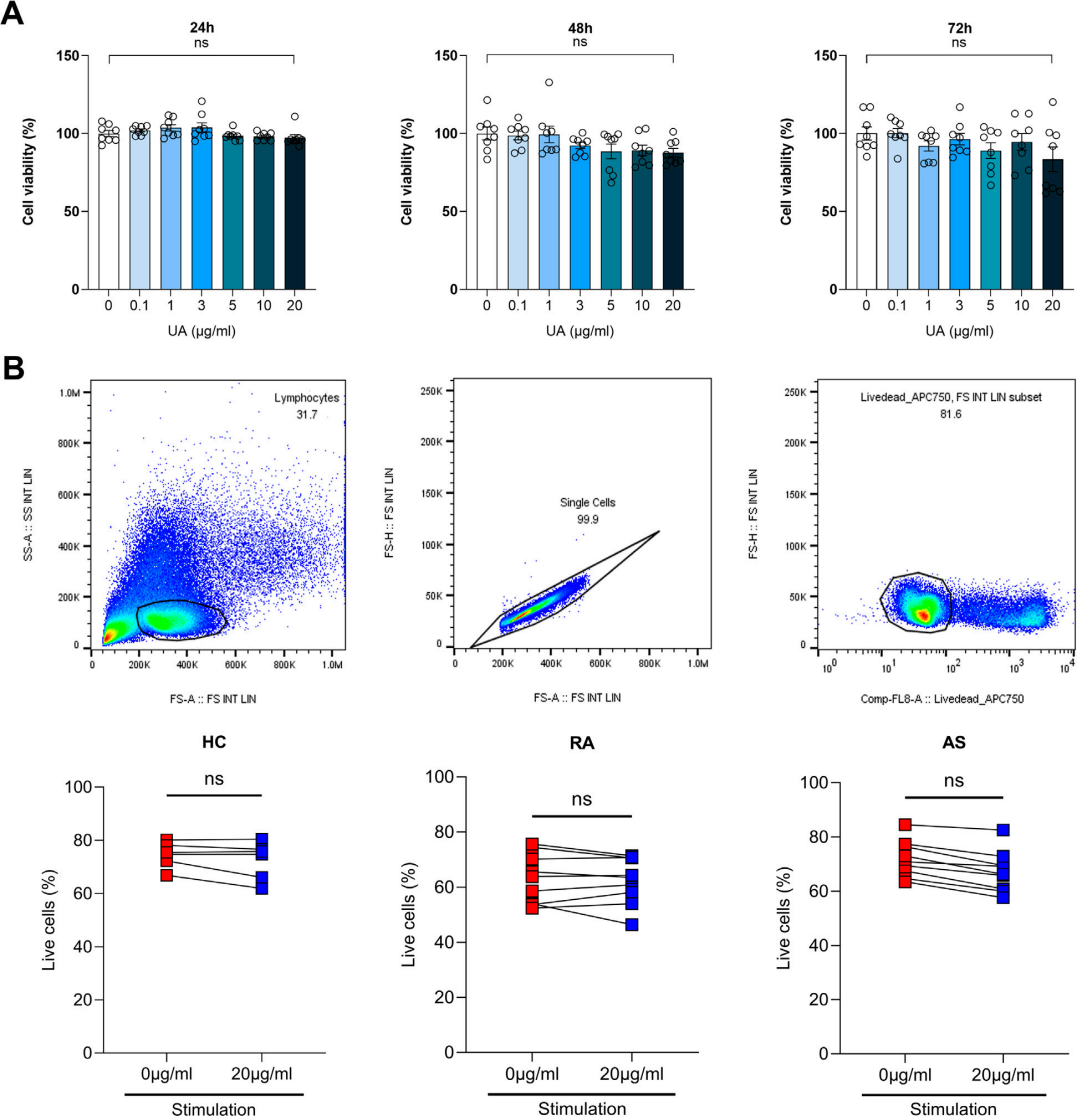
Assessment of cell viability upon UA treatment. **(A)** Cell viability was measured using MTS analysis for each UA concentration in PBMCs. Kruskal–Wallis test with Dunn’s multiple comparisons was performed to determine statistical significance. Values are the mean ± SD. **(B)** HC, RA and AS PBMCs cell viability was measured by flow cytometry. Viability dyes of PBMCs were stained and measured. Mann-Whitney test was performed to determine statistical significance. Values are the mean ± SD. Symbols represent the individual sample. UA, Usenamine A; HC, Healthy Control; RA, Rheumatoid Arthritis; AS, Ankylosing Spondylitis; ns, not significant.

In this study, we investigated the impact of UA on cell viability across various concentrations and time points, utilizing peripheral blood mononuclear cells (PBMCs) from healthy controls (HC), as well as from patients with RA and AS. The assessment of UA’s effects on cell survival was meticulously conducted using the MTS assay, a well-established method for evaluating cell metabolic activity as an indicator of viability. PBMCs were exposed to a range of UA concentrations, specifically 0.1, 1, 3, 5, 10, and 20 μg/mL, over three distinct incubation periods: 24, 48, and 72 h (Figure 2A). The findings from the MTS assay revealed that UA did not significantly impact the survival of HC PBMCs across all tested concentrations and time points, suggesting that UA possesses a favorable safety profile with regard to cellular viability. To further validate these findings and to expand the scope of the investigation, cell viability was also assessed using flow cytometry. Flow cytometry analysis was performed on PBMCs from health controls, as well as from individuals diagnosed with RA and AS, providing a broader understanding of UA effects on cell survival within a pathological context. The results corroborated the observations from the MTS assay, indicating that UA did not adversely affect cell viability in PBMCs from either health controls or patients with RA and AS (Figure 2B).

### 3.3 UA harbors inhibitory effects on inflammatory cytokines in human PBMCs

**FIGURE 3:**
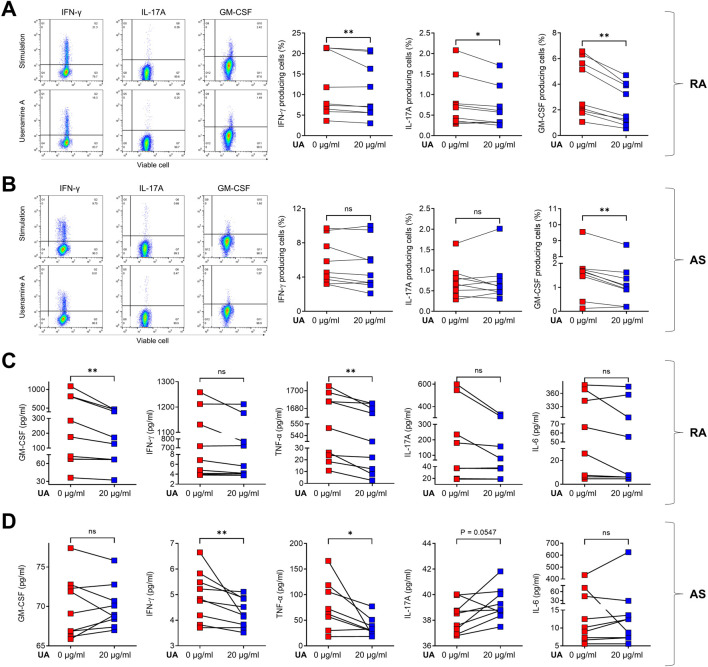
UA treatment reduces cytokine production in RA and AS PBMCs. Cells were pre-treated for 3 h in the presence (red squares) or absence (blue squares) of UA 20 μg/mL treatment, and then cells were stimulated with CD3/CD28 for 24 h. Cells secreting IFN-γ, IL-17A, and GM-CSF were analyzed using flow cytometry for **(A)** RA PBMCs and **(B)** AS PBMCs. Supernatants from UA-treated **(C)** RA and **(D)** AS PBMCs were analyzed using the ELISA to measure GM-CSF, IFN-γ, IL-17A, TNF-α, and IL-6. A Wilcoxon matched pairs signed rank test was performed to determine statistical significance. Values are mean ± SD. Symbols represent the individual sample. UA, Usenamine A; RA, Rheumatoid Arthritis; AS, Ankylosing Spondylitis; ns, not significant; * *P* < 0.05. ** *P* < 0.01.

Following the initial determination that UA did not compromise cell viability, a subsequent phase of the study was initiated, focusing on the immunomodulatory effects of UA on PBMCs derived from patients with RA and AS. In this study, we aimed to provide sufficient time for the treatment of UA on human PBMCs. A duration of more than 24 h was necessary to allow all immune cells to adequately respond to UA, with observations conducted 24 h post-treatment. We utilized CD3/CD28 stimulation, which enables stable activation of immune cells over this 24-hour period. This approach not only directly stimulates T cells but also indirectly activates other immune cell types present in the PBMC population. The FACS analysis revealed a notable reduction in the levels of several critical immune cytokines following UA treatment. Specifically, in the PBMCs from RA patients, there was a marked decrease in the production of interferon-gamma (IFN-γ), interleukin-17A (IL-17A), and granulocyte-macrophage colony-stimulating factor (GM-CSF), as illustrated in Figure 3A. Similarly, in the PBMCs from AS patients, a significant reduction in GM-CSF levels was observed, as depicted in Figure 3B. To further elucidate the effects of UA on cytokine production, a co-culture system was employed wherein the supernatant from the treated PBMCs was subjected to UA exposure. The levels of immune cytokines in the supernatant were then quantitatively measured using enzyme-linked immunosorbent assay (ELISA). In the context of RA, there was a pronounced decrease in the levels of GM-CSF and tumor necrosis factor-alpha (TNF-α), indicating a substantial reduction in pro-inflammatory cytokine production (Figure 3C). Similarly, in AS, the levels of IFN-γ and TNF-α were significantly reduced, as shown in Figure 3D.

### 3.4 UA attenuates inflammatory disease course in an arthritis mouse model

**FIGURE 4:**
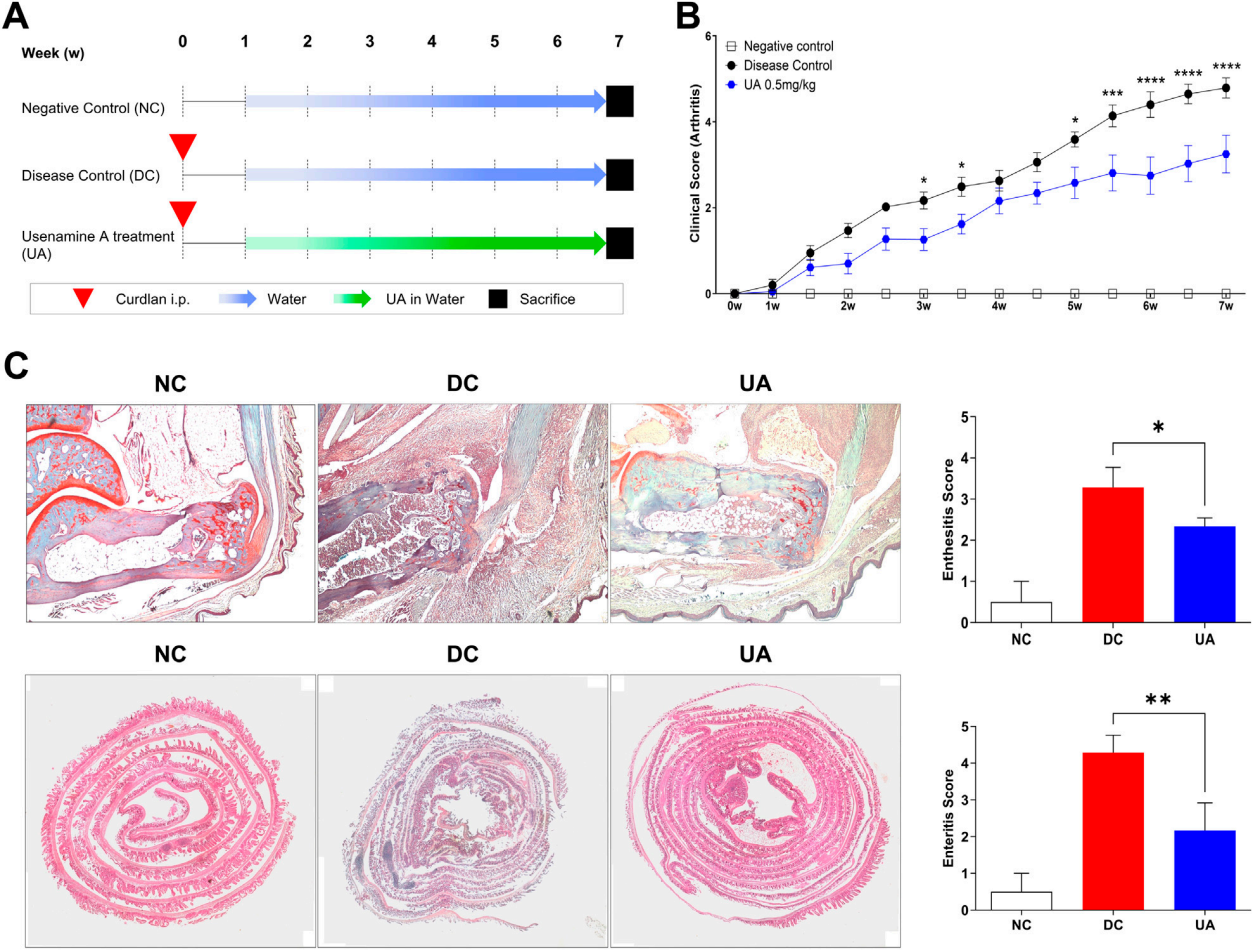
UA treatment reduces clinical scores in SKG mice. **(A)** SKG mice were injected with curdlan i.p. After 1 week, treatment was performed with water or water with UA for 6 weeks. **(B)** Clinical scores were determined according to arthritis severity. Two-Way ANOVA with Dunnett multiple comparisons was performed to determine statistical significance. **(C)** Ankle was stained with Safranin O, the intestine was stained with H&E, and the degree of inflammation was analyzed. Mann-Whitney test was performed to determine statistical significance. Values are the mean ± SEM. Symbols represent the individual sample. * *P* < 0.05, ** *P* < 0.01, *** *P* < 0.001, **** *P* < 0.0001.

In an effort to explore the anti-inflammatory properties of UA within *in vivo* settings, our study employed mouse models subjected to induced inflammation. This phase of the research was initiated 1 week after the administration of curdlan to induce an inflammatory state within the mice. The experimental setup was designed to compare the outcomes between three groups: one receiving standard water and the other receiving water supplemented with UA at a dosage of 0.5 mg/kg, as depicted in Figure 4A. In the context of our study, which involved administering UA dissolved in water to mouse models, it was imperative to verify the adequacy of UA intake compared to the control group, which received water without UA. To address this, our research team meticulously analyzed the water consumption rates across groups. The analysis was conducted by measuring the volume of water consumed by each group over a specified period, allowing for an accurate assessment of intake. Remarkably, the data revealed no significant differences in the amount of water consumed between the groups. This equivalence in water intake across the groups substantiated the conclusion that the differential impacts observed in the study were indeed attributable to the presence of UA in the treatment group’s water, rather than disparities in fluid consumption (Supplementary Table S1).

The evaluation of clinical scores served as a quantitative measure of the inflammatory response and overall clinical condition of the mice. Remarkably, data analysis revealed a significant reduction in clinical scores among the mice treated with UA, indicating a pronounced alleviation of the induced inflammatory state (Figure 4B). This reduction in clinical scores not only underscores the potential of UA as an anti-inflammatory agent but also sets the stage for further histopathological examination to discern the specific impacts at the tissue level. The findings from these examinations were compelling, revealing a noticeable decrease in the degree of enthesitis within the ankle tissue and enteritis within the intestinal tissue of mice in the UA treatment group (Figure 4C). These histopathological outcomes not only corroborate the clinical scoring data but also illuminate the specific anti-inflammatory effects of UA at a tissue level.

## 4 Discussion

This study is the first to underscore the potential of UA as a therapeutic agent capable of modulating immune responses in inflammatory rheumatic diseases. By significantly decreasing the production of pro-inflammatory cytokines in both RA and AS patient-derived PBMCs, UA demonstrates promising anti-inflammatory properties. By showing a marked reduction in tissue inflammation in the UA-treated mice, our study highlights the potential of UA as a therapeutic agent capable of attenuating inflammatory responses in mouse models. The significance of these findings lies not only in their contribution to the understanding of UA’s anti-inflammatory mechanisms but also in their potential implications for developing novel treatments for inflammatory diseases based on UA’s properties.

The bioactive chemical components of *Usnea* encompass Usnic acid, barbatic acid, diffractaic acid, ramalic acid, lichenin, and ethyl everninate (Bao and Bau, 2013). These constituents contribute to the myriad pharmacological activities exhibited by *Usnea*, as indicated by previous studies. Its therapeutic potential extends to antimicrobial properties (Nishanth et al., 2015), antioxidant effects (Singh et al., 2016), antitumor activity (Yang et al., 2016), and anti-inflammatory effects (Vanga et al., 2017). It has even found application in weight reduction (Prateeksha et al., 2016). The wealth of beneficial properties associated with *Usnea* underscores its significance as a source of bioactive compounds with diverse medicinal applications. First of all, we aimed to find evidence regarding on anti-inflammatory properties of UA through molecular scope. TNF complex demonstrated a high affinity for the active ingredients of UA. These interactions assisted in the understanding of anti-arthritic activity. These data lead us to demonstrate the anti-inflammatory potential of UA in targeting RA or AS. Similarly, Huang et al. previously presented compelling evidence supporting the anti-inflammatory properties of *Usnea diffracta* (Zhijun Huang et al., 2014). Their research demonstrated a reduction in the production of pro-inflammatory factors, including TNF-α, IL-1β, IL-6, and NO, following treatment with usnic acid. The observed effects were confirmed at both the cellular transcriptional and translational levels, aligning with prior findings and emphasizing the potential role of *Usnea diffracta* in mitigating inflammatory diseases. Usnic acid, a key component of *Usnea diffracta*, may exert its anti-inflammatory effects by possibly impacting COX-1 or COX-2, suppressing NF-kB, and elevating anti-inflammatory factors such as IL-10 and HO-1 (Zhijun Huang et al., 2014; Galanty et al., 2021). Notably, the anti-inflammatory potential of usnic acid was further assessed in a rat model with induced chronic and acute inflammation. Collectively, we could give evidence of the utility of UA, one of derivatives of *Usnea diffracta*, which may possess anti-inflammatory potential by integrating TNF complex.

Until now, UA is still unraveled regarding on its pharmacological effect. We confirm that UA is safe and can reduced the production of IFN-γ, IL-17A, and GM-CSF production in PBMC obtained from RA or AS patients. Of note, we consistently observed a reduction of GM-CSF production in PBMC obtained from RA and AS patients. It is well known that GM-CSF plays an important role in inflammatory responses in autoimmune disease via induction of various cells and mediators (Lotfi et al., 2019). Previous studies have reported that GM-CSF potentiates the production of TNF by LPS-stimulated whole blood, PBMCs and monocytes, and further neutralizing GM-CSF significantly reduces the production of TNF in AS patients (Shi et al., 2020). Taken together, these observations support of the potential of UA through GM-CSF neutralization, as a novel therapeutic approach for the treatment of RA or AS.

It is noteworthy that UA treatment group presented ameliorated disease-specific symptoms, including enthesitis and enteritis. Our data suggest that UA treatment can attenuate the symptom severity of RA or AS across all extents of diseases presentations. Although the exact mechanism remains unknown, our data highlight that UA may actively inhibit inflammation of RA or AS, which might be accompanied by attenuation of GM-CSF, IL-17A or TNF.

Our study has several limitations. UA demonstrates anti-inflammatory effects by docking to the TNF complex. However, further research is necessary to elucidate the specific mechanisms of UA’s anti-inflammatory actions post-docking. Additionally, more samples from RA and AS patient are needed to further confirm the anti-inflammatory effects.

Our preliminary data indicate that UA treatment was effective in suppressing inflammatory cytokines in RA and AS patients and was also effective in alleviating arthritis in a mouse model. Our findings suggest potential effectiveness in the treatment of rheumatic diseases.

## Data Availability

The original contributions presented in the study are included in the article/Supplementary Material, further inquiries can be directed to the corresponding authors.
