# Supplementary figures and images for: Usenamine A: a potential therapeutic agent for rheumatoid arthritis and ankylosing spondylitis through its anti-inflammatory activity
Source: Front Pharmacol. 2024 Dec 3;15:1456216. doi: 10.3389/fphar.2024.1456216 (PMC11650205; doi:10.3389/fphar.2024.1456216)

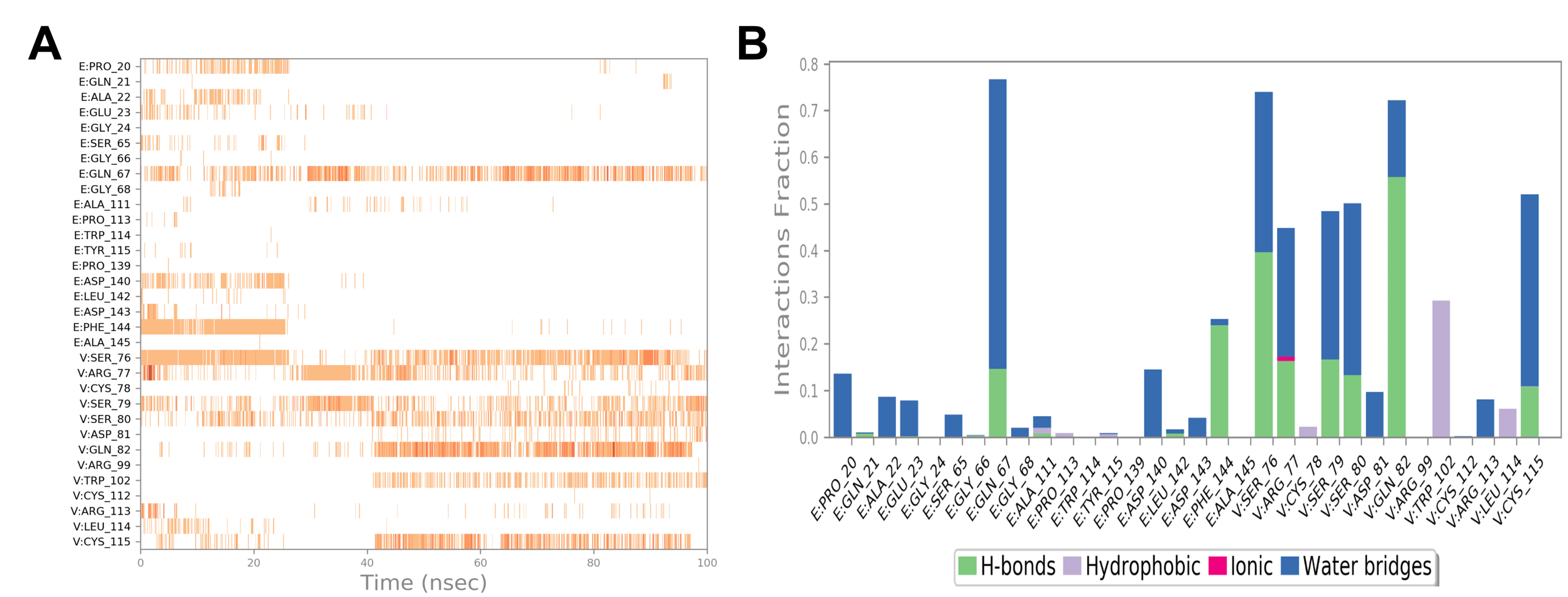

Supplement: Supplementary file 1 [file Image1.jpg]
