# Supplementary material for: Usenamine A: a potential therapeutic agent for rheumatoid arthritis and ankylosing spondylitis through its anti-inflammatory activity
Source: Front Pharmacol. 2024 Dec 3;15:1456216. doi: 10.3389/fphar.2024.1456216 (PMC11650205; doi:10.3389/fphar.2024.1456216)
Supplement: Supplementary file 2 [file DataSheet1.docx]

Supplementary Material

# Supplementary Figures and Tables

## Supplementary Tables

|  | | | Drink water (ml) | |
| --- | --- | --- | --- | --- |
|  | | | Positive control | UA 0.5mg/kg |
|  | 1 week | amount of drink | 120 | 105 |
|  |  | per mouse | 3.43 | 3.00 |
| water or UA in water | 2 weeks | amount of drink | 125 | 135 |
|  |  | per mouse | 3.57 | 3.86 |
| water or UA in water | 3 weeks | amount of drink | 155 | 130 |
|  |  | per mouse | 4.43 | 3.71 |
| water or UA in water | 4 weeks | amount of drink | 135 | 130 |
|  |  | per mouse | 3.86 | 3.71 |
| water or UA in water | 5 weeks | amount of drink | 125 | 130 |
|  |  | per mouse | 3.57 | 3.71 |
| water or UA in water | 6 weeks | amount of drink | 125 | 130 |
|  |  | per mouse | 3.57 | 3.71 |
| water or UA in water | 7 weeks | amount of drink | 125 | 135 |
|  |  | per mouse | 3.57 | 3.86 |
| Average (ml) | | | 3.71 | 3.65 |

**Supplementary Table 1.** The amount of water consumed by mice was measured in groups from week to week.

## Supplementary Figures


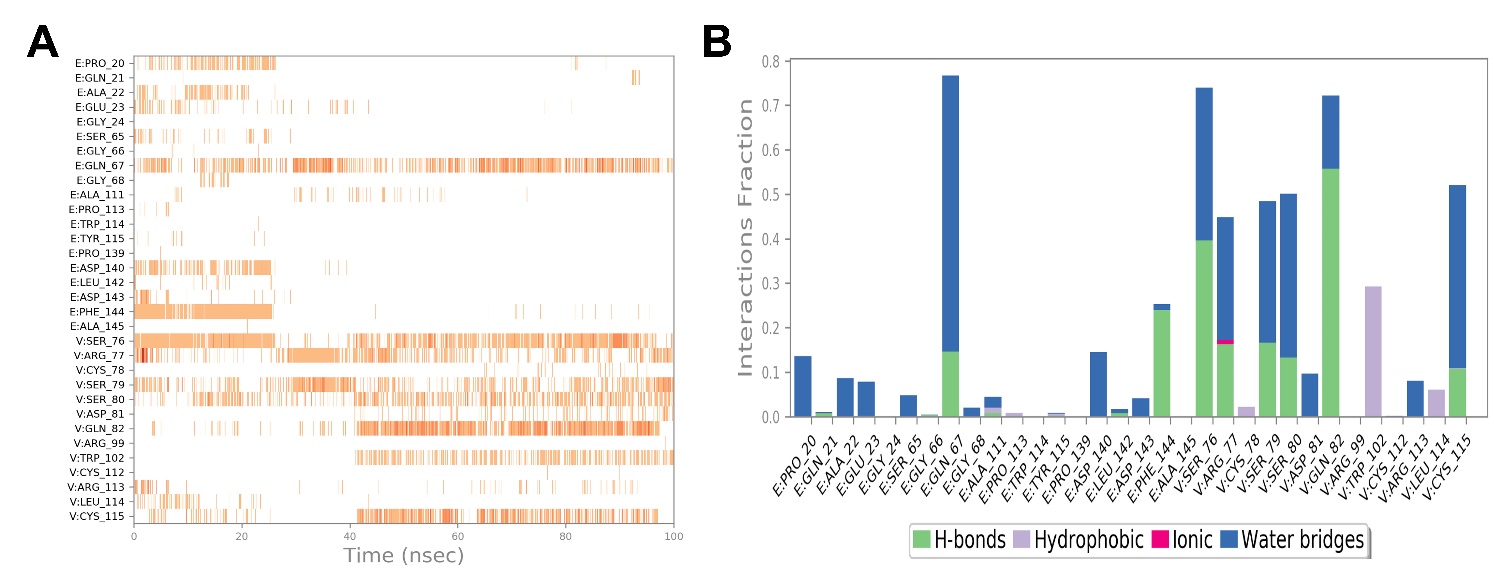


**Supplementary Figure 1.** (A**)** Interaction shown by the active site amino acids in each trajectory of usenamine A-3ALQ, and (**B)** the histogram of usenamine A-3ALQ interaction over the period of the trajectory.
